# Does the Correspondence Bias Apply to Social Robots?: Dispositional and Situational Attributions of Human Versus Robot Behavior

**DOI:** 10.3389/frobt.2021.788242

**Published:** 2022-01-04

**Authors:** Autumn Edwards, Chad Edwards

**Affiliations:** Communication and Social Robotics Labs, School of Communication, Western Michigan University, Kalamazoo, MI, United States

**Keywords:** fundamental attribution error, correspondence bias, social robot, human-robot interaction, computers are social actors, behaviorism

## Abstract

Increasingly, people interact with embodied machine communicators and are challenged to understand their natures and behaviors. The Fundamental Attribution Error (FAE, sometimes referred to as the correspondence bias) is the tendency for individuals to over-emphasize personality-based or dispositional explanations for other people’s behavior while under-emphasizing situational explanations. This effect has been thoroughly examined with humans, but do people make the same causal inferences when interpreting the actions of a robot? As compared to people, social robots are less autonomous and agentic because their behavior is wholly determined by humans in the loop, programming, and design choices. Nonetheless, people do assign robots agency, intentionality, personality, and blame. Results of an experiment showed that participants made correspondent inferences when evaluating both human and robot speakers, attributing their behavior to underlying attitudes even when it was clearly coerced. However, they committed a stronger correspondence bias in the case of the robot–an effect driven by the greater dispositional culpability assigned to robots committing unpopular behavior–and they were more confident in their attitudinal judgments of robots than humans. Results demonstrated some differences in the global impressions of humans and robots based on behavior valence and choice. Judges formed more generous impressions of the robot agent when its unpopular behavior was coerced versus chosen; a tendency not displayed when forming impressions of the human agent. Implications of attributing robot behavior to disposition, or conflating robot actors with their actions, are addressed.

## 1 Introduction

The Fundamental Attribution Error (FAE) is the tendency for people to over-emphasize dispositional or personality-based explanations for others’ behavior while under-emphasizing situational explanations (Ross, 1977). In other words, people sometimes demonstrate a cognitive bias by inferring that a person’s actions depend on what “kind” of person they are rather than on the social and environmental forces that influence the person. As such, an observer will likely attribute reasons for a behavior to internal characteristics and not external factors (Gilbert and Jones, 1986). Individual behavior is heavily influenced and guided by situational and external factors. However, “because people are accustomed to seeing individuals as causal agents, viewing the actor and (their) actions as forming a single categorical unit also appears to be the simplest, most satisfying, and least effortful inferential strategy (Heider and Simmel, 1944; Heider, 1958; Jones, 1979)” (Forgas, 1998, p. 319).

Although this effect has been thoroughly examined with humans, we do not know if the same correspondence bias will apply to social robots. When communicating with machines such as social robots, people must form impressions of the agents and judge their behavior. Compared to people, current robots are less agentic and autonomous with behaviors driven by programming, design, and humans in the loop. However, people do nonetheless assign robots agency, intentionality, and blame (Sciutti et al., 2013; De Graaf and Malle, 2019; Banks, 2020). The purpose of this experiment is to determine whether people commit the FAE in response to the behaviors of a social robot. FAE is sometimes referred to as the correspondence bias (Gawronski, 2004), an issue we will return to in the discussion. Whereas the FAE assumes a general tendency to underestimate the power of situation on human behavior, the correspondence bias refers more narrowly to the tendency to make disposition-congruent inferences of observed behavior. However, because much of the literature uses both FAE and correspondence bias, we will use the terminology cited in the mentioned studies in the next sections.

## 2 Fundamental Attribution Error

Research has demonstrated that the FAE may distort an observer’s judgment of an individual, especially in the case of overattribution of individual responsibility for large achievements or grave mistakes (Ross et al., 1977). Previous research has demonstrated that individuals who commit the FAE assign too much personal responsibility for both positive and negative outcomes (Ross et al., 1977; Riggio and Garcia, 2009). According to research on the FAE, individuals use two types of information when making attributions: dispositional and situational (Pak et al., 2020). As such, the FAE “rests on an assumption of dualism: that there is a clear division between what is inside and outside the person” (Langdridge and Butt, 2004, p. 365).

Dispositional attributions pertain to perceived qualities of the individual, whereas situational attributions pertain to perceived characteristics of the environment and factors outside of the individual’s control. “Potential biases in the causal attribution process can come from the valence of the situational outcome (was the outcome positive or negative), the degree of informational ambiguity of the situation, and the degree of control an actor has over an outcome” (Pak et al., 2020, p. 422). FAE has been examined in relation to behavioral judgments. For example, when presented with an excerpt of a character’s bad day, students tended to attribute the cause to dispositional versus situational factors (Riggio and Garcia, 2009). However, students who were primed by watching a video about the power of social and environmental influences on individual behavior attributed the cause of the bad day more to situational factors. Therefore, broader construal may help attenuate the FAE.

FAE does not seem to be universal across cultures but does exist heavily in Western cultures (Norenzayan and Nisbett, 2000). Research in social psychology has forwarded several explanations for why individuals commit the FAE. The first explanation is that people are more likely to attribute causes or responsibilities to an observed than an unobserved element. Because agents are more salient than their situations in many judgment tasks, the agent itself draws observers’ attributional focus (Taylor and Fiske, 1975; Robinson and McArthur, 1982). The second explanation is that personal/dispositional attributions are more comforting causal inferences because they reinforce the just-world hypothesis, which holds that “people get what they deserve” or “what goes around comes around” (Walster, 1966). However, this explanation better explains deliberative judgments than the swift or automatic judgments often formed in response to individual behavior (Berry and Frederickson, 2015).

The third explanation is that humans may have evolved (and learned) to be hypersensitive in terms of agency detection. HADD, or the hypersensitive agency detection device, is the cognitive system theorized to be responsible for detecting intentional agency (Barrett, 2000). People overestimate the presence of human agency and therefore demonstrate a bias in which situations and events are attributed to people or other human-like entities. Agency detectors are so sensitive that even movement is enough to trigger attributions of will and intention, as evidenced in a number of Theory of Mind (ToM) studies (Barrett, 2007).

### 2.1 Attributional Process in Human-Robot Interaction

People attribute mental states to others in order to understand and predict their behavior. There is evidence of similarity in how people interpret humans’ and robots’ actions in the sense that people implicitly process robots as goal-oriented agents (Sciutti et al., 2013), use the same “conceptual toolbox” to explain the behavior of human and robot agents (De Graaf and Malle, 2019), make implicit Theory of Mind (ToM) ascriptions for machine agents (Banks, 2020), and evaluate a social robot’s message behavior in terms of its underlying beliefs, desires, and intentions for communication (Edwards et al., 2020). HRI scholars have argued that the physical presence of a robot, or embodied machine agent, can produce patterns of attributions similar to those occurring in human-human interaction (Ziemke et al., 2015; De Graaf and Malle, 2017; Pak et al., 2020). Even when participants were provided with transparent information about how a robot makes decisions, they still attributed outcomes of behaviors to robot thinking (Wortham et al., 2017), which suggests the persistence of dispositional attributions even when situational information is provided (Pak et al., 2020). In addition, people have been found to use folk-psychological theories similarly to judge human and robot behavior in terms of ascriptions of intentionality, controllability, and desirability and in the perceived plausibility of behavior explanations (Thellman et al., 2017). Furthermore, there is evidence that human-linked stereotype activation (e.g., stereotypes of aging) influences causal attributions of robot behavior (Pak et al., 2020). The results of such studies generally lend support to the Computers are Social Actors (CASA) paradigm, which posits that people tend to treat and respond to machine agents with social cues in the same ways they do other people (Reeves and Nass, 1996).

The Form Function Attribution Bias (FFAB) refers to cognitive shortcuts people take based on the robot’s morphology or appearance (Haring et al., 2018). The FFAB leads people to make biased interpretations of a robot’s ability and function based on the robot’s physical form (Hegel et al., 2009) and the perceived age of the robot (Branyon and Pak, 2015). Some research has demonstrated that attributions of action and mind increased as more human features were included in pictures of robot/avatar faces (Martini et al., 2016). Interacting with robotic agents on a task reduced one’s own sense of agency similar to working with other individuals (Ciardo et al., 2020). This effect was not observed with non-agentic mechanical devices. Other research suggests that agent-category cues help shape perceptions which then influence behavioral outcomes (Banks et al., 2021). In doing so, there is a tendency to judge action on the basis of the agent performing it. Although these findings do not speak directly to the applicability of the FAE to social robots, they do demonstrate that attributional patterns similar to those observed in human interaction may emerge when people interact with social machines.

As a result, it is important to understand how the FAE may impact perceptions of a social robot when the robot engages in popular or unpopular behavior. These findings will have implications for how humans understand the causes of social robots’ behavior and assign blame or credit for their activities, which is increasingly relevant in contexts including emergency/crisis, healthcare, education, retail, and legal. In short, how will people assign the cause of a robot’s behavior in relation to how they do so for other humans? More specifically, to closely replicate the experimental research on FAE in human interaction (Forgas, 1998), we will focus on a situation in which a robot or human expresses the popular or unpopular position on a topic of social importance. This design falls within the attributed attitude paradigm of research investigating the correspondence bias (Jones and Harris, 1967). Although application of the CASA paradigm would suggest people will demonstrate similarity in their attributional processes of human and robot behavior, the observed differences in people’s responses may indicate differences. The traditional procedure for carrying out research within the CASA framework entails 1) selection of a theory or phenomenon observed in human interaction, 2) adding humanlike cues to a robot, 3) substituting the robot for a human actor, and 4) determining whether the same social rule applies (Nass et al., 1994). To also allow for identification of more granular potential differences in how people respond to robots, the present study modifies and extends the procedure to include a human-to-human comparison group. We offer the following research questions:

RQ1: Will participants attribute the cause of an agent’s (social robot or human) behavior to disposition or situational factors?

RQ2: How will the nature of an agent’s behavior (popular or unpopular) influence attributions and impressions?

## 3 Materials and Methods

### 3.1 Participants

The sample included 267 U.S. American adults recruited and compensated US $2.00 through Amazon’s Mechanical Turk. Participants who 1) failed the audio test, 2) failed the speech-topic attention check or 3) reported non-normative attitudes toward the topic (opposed legalization of medicinal marijuana) were excluded from analysis, leaving 231 participants. Their average age was 43.32 years (*SD* = 11.36, *MD* = 40, *range* = 24–71). Slightly over half identified as male (51%, *n* = 118), followed by female (48%, *n* = 110), those who selected “prefer to not answer” (0.9%, *n* = 2), and gender fluid (0.4%, *n* = 1). Predominantly, participants identified as White (79%, *n* = 183), followed by Black or African-American (7%, *n* = 16), Hispanic or Latino/a/x (5%, *n* = 12), Asian or Pacific Islander (5%, *n* = 12), bi- or multi-racial (3%, *n* = 7), and one person (0.4%) selected “prefer to not answer”. Most had a Bachelor’s degree or higher (60%, *n* = 138).

### 3.2 Procedures

Procedures entailed a modified replication of Forgas’ (1998) experiments investigating the correspondence bias by examining the degree to which people attributed a person’s message behavior to their “true attitudes” about the topic when that behavior was popular (normative, and therefore expected) or unpopular, and chosen or coerced (Forgas, 1998). Additionally, Forgas manipulated the mood of participants as happy or sad to determine the influence of mood on attributional judgements. Participants were asked to read an essay forwarding either a popular or unpopular position on the topic of French nuclear testing in the South Pacific, which was framed as either the chosen stance of the author or an assigned/coerced stance. Then, participants were asked to consider whether the essay represented the true attitude of its writer, to indicate their degree of confidence in that attribution, and to give their impressions of the essay writer. In the present study, we replicated the basic design with four modifications: 1) manipulation of the agent as human or robot, 2) use of a more contemporary topic (medicalization of marijuana), 3) speeches versus essays, and 4) measured and statistically controlled for mood rather than manipulated mood.

Upon securing Institutional Review Board approval and obtaining informed consent, we conducted a 2 (agent: human vs. robot) X 2 (behavior: popular vs unpopular) X 2 (choice: chosen vs. coerced) between-subjects online video experiment, which was introduced to participants as a “social perception study.” After completing an audio check, participants were asked to rate their current affective/mood state. Next, participants were randomly assigned to view one of eight experimental conditions involving a 1-min video containing a persuasive appeal by a human or a robot, in which the agent advocated for or against legalizing medical marijuana (operationalizing popular vs. unpopular behavior), with the position stipulated as either freely chosen by or assigned to the speaker. As a manipulation and attention check, participants were asked to report the speaker’s stated position in the video before progressing to the rating tasks. Next, they were asked a series of questions assessed along 7-point semantic differential scales to ascertain 1) inferences of the speakers’ “true attitudes” toward legalizing medical marijuana, 2) confidence in their attributed attitude ratings, and 3) interpersonal impressions of the speaker. Finally, they were asked to report their own attitudes toward the legalization of medical marijuana, to offer any open-ended comments, and to provide demographic information.

### 3.3 Mood Check

Prior to the experimental task, participant mood was self-assessed with two (1:7) semantic differential items rating current mood as sad:happy and bad:good. Answers were highly related [*r* (228) = 0.92, *p* < 0.001] and therefore summed to create a single mood score, *alpha* = 0.96, *item M* = 5.98, *SD* = 1.40.

### 3.4 Attribution Task

Participants were asked to “carefully watch a 1-min persuasive speech written by this (person/robot),” who, they were informed, either chose to take this stance on the issue (choice) or was assigned to do so (coerced). Next, they were asked to answer a series of questions about the speaker. The speeches dealt with the familiar and salient topic of the legalization of marijuana for medical purposes. There is a strongly preferred normative position on the issue, with 91% of U.S. Americans in favor of legalization for medical use [59% for medical and recreational +32% for medical use only; (Daniller, 2019)]. The speeches persuading for and against legal marijuana (popular and unpopular behavior, respectively) were identical except for single phrases or words substituted to reverse the sentiment and meaning of the two parallel conditions. For example, “Medical marijuana should (should not) be legal,” “Legalizing medical marijuana is (is not) in the public’s best interest” and “Legal medical marijuana will (will not) be effectively regulated for consumers.” The overall position forwarded in each speech was clearly and strongly for or against legalization.

### 3.5 Agent Manipulation

For the human agent conditions, a graduate research assistant unknown to participants delivered the 1-min persuasive appeal for and against legalization. The robot agent was Softbank’s Pepper humanoid robot, which was programmed to deliver the same scripted speeches with a matching rate of speech and comparable animacy of gestures and movement. For both the human and robot conditions, the video frame included the face and upper body in front of a light-colored backdrop.

### 3.6 Dependent Variables

After watching the speeches, participants rated the speaker along a series of 7-point bipolar scales, which assessed 1) perceptions of the speaker’s “real attitudes” toward the issues (“What do you think the speaker truly believes about legalizing medicinal marijuana?” Supports it–Opposes it), 2) levels of confidence in attributed attitudes (“How confident are you in knowing what the speaker truly believes about the issues?” Confident–Not Confident), and 3) global impressions of the speaker (Dislikable–Likable; Unpopular–Popular: Unintelligent–Intelligent; Incompetent–Competent; Untrustworthy–Trustworthy; Inexpert–Expert; Uncaring–Caring; Unsimilar–Similar), with items similar to those used to in previous studies of the correspondence bias e.g., (Forgas, 1998).

### 3.7 Attitude Assessment

Participants’ attitudes toward the issue of legalizing medical marijuana was also assessed. Approximately 92% of the sample supported the position that “medical marijuana should be legal,” which indicated the strong popularity of the pro-legalization speech stances. As noted above, potential participants who opposed the legalization of medical marijuana were subsequently excluded from analysis to ensure that pro-legalization speeches operationalized “popular” behavior [i.e., a strongly preferred normative and therefore probabilistic opinion; (Jones and Harris, 1967)].

## 4 Results

### 4.1 Mood

Participants’ mood states at the beginning of the experiment were statistically controlled as a covariate in all analyses because mood has been found to influence the degree to which judges demonstrate the correspondence bias. Specifically, happy mood enhanced and sad mood lessened dispositional attributions of coerced unpopular behavior (Forgas, 1998).

### 4.2 Attribution of Attitude to the Speaker

A three-way analysis of covariance (ANCOVA) evaluated the effects of agent (human vs. robot), behavior (popular vs. unpopular), and choice (chosen vs coerced) on attributed attitudes while controlling for mood. See Table 1 for means and standard deviations.

**TABLE 1:** Means and standard deviations for attributed attitudes (1–7; opposes it:supports it).

				Behavior	
		Popular		Unpopular		Total	
Agent	Choice	M	SD	M	SD	M	SD
Human	Chosen	5.72	1.12	3.81	1.94	4.86	1.83
	Coerced	5.31	1.04	4.00	1.94	4.81	1.57
Robot	Chosen	5.91	1.23	2.62	1.60	4.29	2.18
	Coerced	5.63	1.35	2.48	1.35	4.08	2.08
Total	Chosen	5.82	1.21	3.16	1.84	4.56	2.03
	Coerced	5.47	1.21	3.06	1.75	4.41	1.90

As expected, in a significant main effect of behavior, agents that expressed popular versus unpopular positions were judged to hold significantly different attitudes about the issue (*M* = 5.65 vs. 3.11), *F* (1, 220) = 152.98, *p* < 0.001, *partial eta squared* = 0.41. There was also a significant main effect of agent with stronger pro-legalization attitudes attributed to the human versus robot (*M* = 4.84 vs. 4.19), *F* (1, 220) = 7.39, *p* < 0.01, *partial eta squared* = 0.03. The two-way interaction between behavior and choice was not significant, *F* (1, 220) = 0.84, *p* = 0.36, *partial eta squared* = 0.00. Differing, topic-congruent attitudes were attributed to agents that expressed popular versus unpopular positions regardless of whether their stances were chosen or coerced, establishing that judges demonstrated a correspondence bias, or FAE, in attributing attitudes.

**FIGURE 1:**
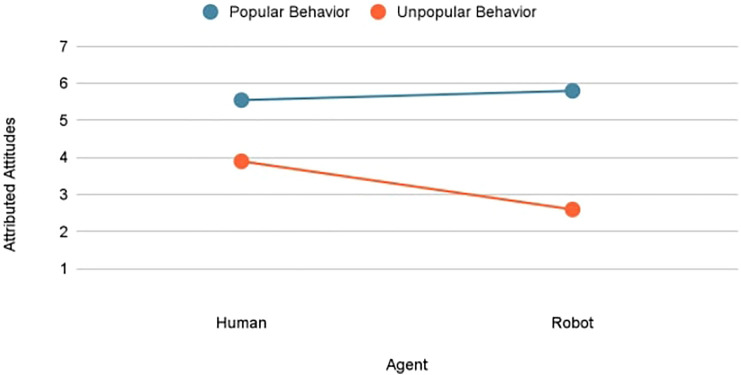
Interaction effect of agent and behavior on attributed attitudes.

As shown in Figure 1, there was a significant interaction between behavior (popular vs. unpopular) and agent (human vs. robot), *F* (1, 220) = 16.15, *p* < 0.001, *partial eta squared* = 0.07. Analysis of simple main effects showed that different attitudes were attributed to agents expressing popular versus unpopular positions in both the human (*M* = 5.52 vs. 3.89) and robot (*M* = 5.78 vs. 2.56) conditions.

**FIGURE 2:**
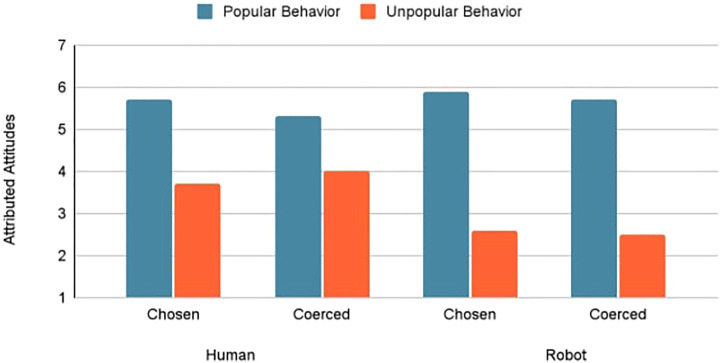
Simple main effects of choice and behavior on attitudes attributed to agents.

As depicted in Figure 2, agent type had no marked influence on attributions of popular behavior (*M* = 5.52 vs. 5.78). With unpopular behavior, however, the judges inferred the robot to have a stronger topic-congruent attitude compared to the human (*M* = 2.56 vs. 3.89).

Results confirmed that judges made correspondent inferences of both human [*F* (1, 100) = 27.12, *p* < 0.001, *partial eta squared* = 0.21] and robot agents [*F* (1, 119) = 161.94, *p* < 0.001, *partial eta squared* = 0.58], by assuming that their true attitudes aligned with their expressed positions. However, the effect size of behavior (popular vs. unpopular) on attributed attitudes was substantially larger for robots than humans. Judges drew a stronger unit relation between the agent and its behavior when evaluating the robot, as further demonstrated by linear regressions treating attributed attitudes as criterion and behavior valence as predictor in human and robot conditions. When judging humans, behavior valence was a significant predictor of attributed attitudes, *Beta* = −0.48, *t* (104) = −5.66, *p* < 0.001, and explained significant variance in attributed attitudes, *adjusted r* ^*2*^ = 0.23, *F* (1, 104) = 32.02, *p* < 0.001. However, behavior valence was a stronger predictor of attitudes attributed to robots [*Beta* = −0.76, *t* (122) = -12.95, *p* < 0.001] and produced a larger effect size [*adjusted r* ^*2*^ = 0.58, *F* (1, 122) = 167.62, *p* < 0.001]. The relatively stronger correspondence bias toward robots was driven by the greater dispositional culpability attributed to robots engaging in unpopular behavior (anti-legalization stance), whether freely chosen or coerced.

### 4.3 Confidence in Attitude Judgments

**FIGURE 3:**
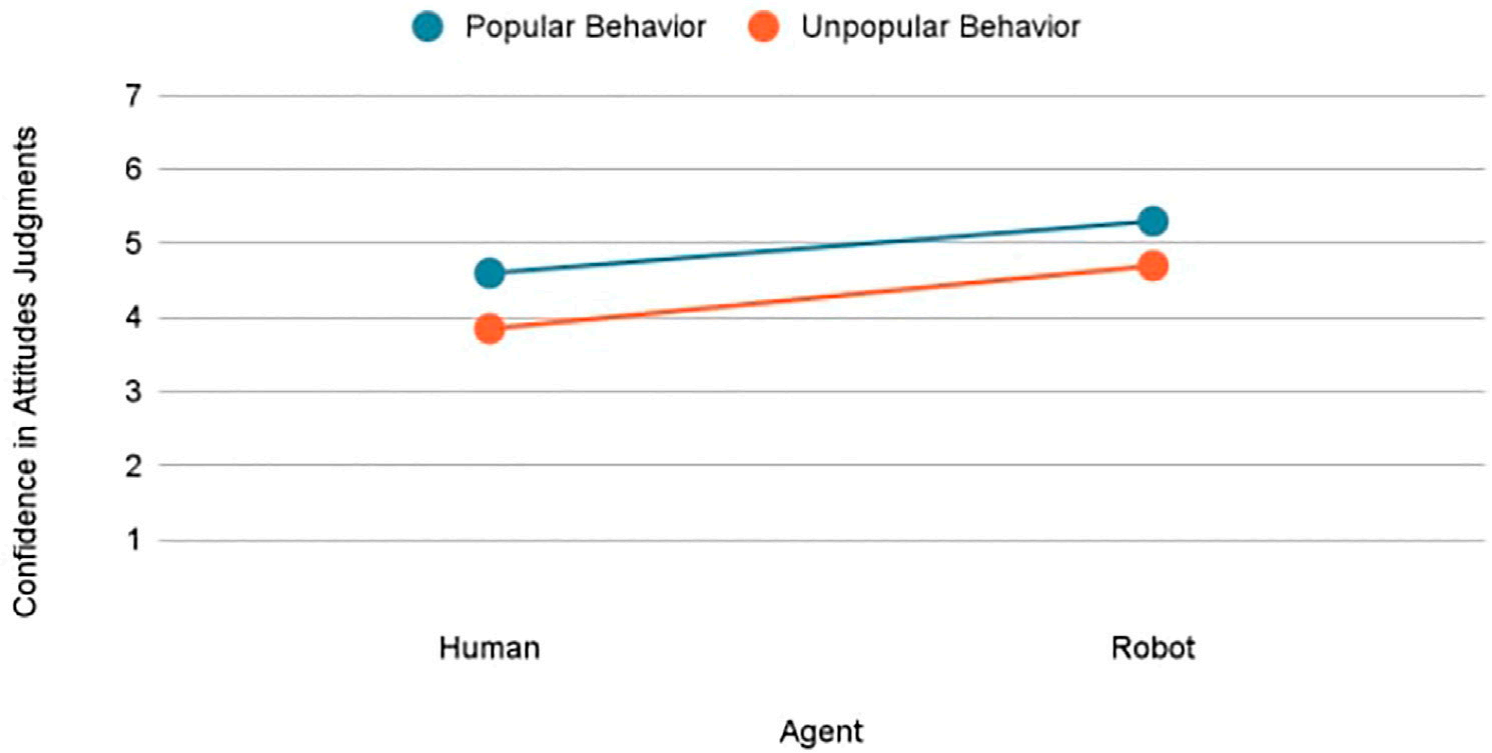
Effects of agents and behavior on confidence in attitude attribution.

Confidence ratings for attitude judgments were analyzed to assess any awareness by judges of their attributional limitations (Figure 3). Choice had no significant influence on confidence in attributions [*F* (1, 220) = 1.71, *p* = 0.193, *partial eta squared* = 0.01]; participants felt equally confident in their attitude attributions of speakers whose positions were chosen versus coerced. Both agent [*F* (1, 220) = 6.02, *p* = 0.015, *partial eta squared* = 0.03] and behavior [*F* (1, 220) = 5.04, *p* = 0.026, *partial eta squared* = 0.02] had a main effect on confidence. Judges reported greater confidence in their attributions of popular versus unpopular positions (*M* = 4.94 vs. 4.42) and of robot versus human agents (*M* = 4.95 vs. 4.42). Judges drew a stronger unit relation between the robot agent and its actions, and also felt greater confidence in their judgments of the robot’s attitudes.

### 4.4 Impressions

Impression judgments on the eight bipolar scales were factor analyzed. Visual inspection of the scree plot and consideration of Eigenvalues > 1.00 supported treatment as unidimensional (Eigenvalue = 5.27; 65.86% variance; highest loading item = unlikable:likable). Therefore, we summed the items to form the impressions dependent variable, *alpha* = 0.92 (*item M* = 5.21, *SD* = 1.45). The effects of agent, behavior, and choice on impressions were assessed with a three-way ANCOVA, again controlling for mood. Table 2 for means and standard deviations.

**TABLE 2:** Means and standard deviations for impressions (1–7; Negative: Positive).

				Behavior	
		Popular		Unpopular		Total	
Agent	Choice	M	SD	M	SD	M	SD
Human	Chosen	6.10	1.01	4.93	1.58	5.56	1.41
	Coerced	5.74	0.94	5.19	1.29	5.53	1.11
Robot	Chosen	5.78	1.26	3.91	1.33	4.86	1.59
	Coerced	5.29	1.58	4.71	1.29	5.00	1.46
Total	Chosen	5.93	1.14	4.38	1.52	5.19	1.54
	Coerced	5.51	1.32	4.89	1.30	5.29	1.34

**FIGURE 4:**
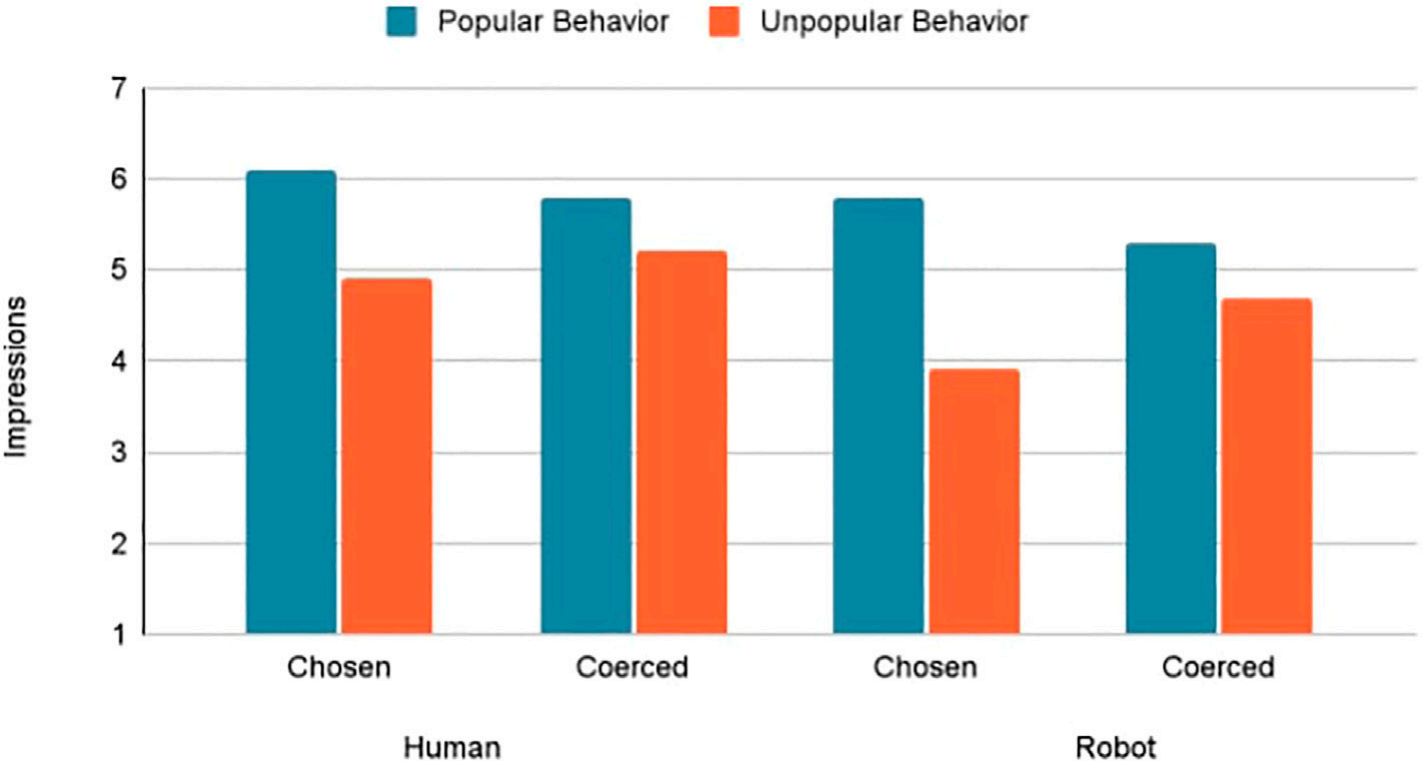
Simple main effects of choice and behavior on impressions of agents.

There was a significant main effect of agent [*F* (1, 221) = 8.75, *p* = 0.003, *partial eta squared* = 0.04] and of behavior [*F* (1, 221) = 38.26, *p* < 0.001, *partial eta squared* = 0.15] with more favorable ratings of humans versus robots (*M* = 5.54 vs. 4.92) and of agents expressing popular versus unpopular positions (*M* = 5.73 vs. 4.60). There was no main effect of choice [*F* (1, 221) = 0.28, *p* = 0.594, *partial eta squared* = 0.001]. However, choice condition and behavior interacted to influence impressions of the agent [*F* (1, 220) = 7.78, *p* = 0.006, *partial eta squared* = 0.03]. Because judges formed different impressions of human and robot agents, simple main effects were examined separately for agent conditions (Figure 4).

#### 4.4.1 Impressions of Human Agent

The human was rated more favorably when taking the popular versus unpopular stance, *F* (1, 101) = 18.40, *p* < 0.001, *partial eta squared* = 0.15, *M* = 5.93 vs. 5.03). There was no significant effect of choice [*F* (1, 101) = 0.052, *p* = 0.820, *partial eta squared* = 0.001] or interaction effect of choice and behavior [*F* (1, 101) = 1.45, *p* = 0.231, *partial eta squared* = 0.014] on interpersonal impressions.

#### 4.4.2 Impressions of Robot Agent

Choice and behavior interacted to influence interpersonal impressions of the robot, *F* (1, 119) = 6.72, *p* = 0.011, *partial eta squared* = 0.05. Robots expressing the popular position garnered the same impressions whether the position was chosen or coerced, *F* (1, 60) = 1.67, *p* = 0.201, *partial eta squared* = 0.019; *M* = 5.78 vs. 5.29. In contrast, a robot expressing the unpopular position was perceived more negatively than a robot coerced to do so (*M* = 3.90 vs. 4.71), *F* (1, 58) = 5.60, *p* = 0.021, *partial eta squared* = 0.09. When the robot’s behavior was freely chosen, the popular stance led to more favorable interpersonal impressions than the unpopular stance, *F* (1, 62) = 32.34, *p* < 0.001, *partial eta squared* = 0.34, *M* = (5.78 vs. 3.91). When the robot’s behavior was coerced, there was no significant difference in the interpersonal impressions formed of popular versus unpopular behavior, *F* (1, 56) = 2.28, *p* = 0.136, *partial eta squared* = 0.04, *M* = 5.29 vs. 4.71). Judges were more generous in their impressions of the robot when its unpopular behavior was coerced rather than chosen; a tendency not displayed when forming impressions of the human agent. Although judges formed different impressions of the robot that chose its position, the direction of coerced position had no marked influence on impressions.

## 5 Discussion

The correspondence bias (FAE) has been thoroughly tested with people, but not with HRI. In general, people tend to overemphasize dispositional explanations for behaviors seen in others and, at the same time, under-emphasize features of the situation (Pak et al., 2020). Because a social robot’s behavior is completely determined by its design, programming, and humans behind the scenes, it is essential to know if people will still commit the correspondence bias for robot behavior. These findings have implications for assigning credit or blame to a social robot’s behaviors. In this section, we will summarize the results, discuss implications, and offer limitations and directions for future research.

### 5.1 Summary of Results

Research question 1 asked if participants would attribute the cause of an agent’s (social robot or human) behavior to disposition or to situational factors. Participants exhibited the correspondence bias (FAE) toward both human and robot agents by assuming their behavior corresponded to their underlying attitudes (a dispositional attribution) even when their behavior was clearly assigned (a situational cause). However, their dispositional correspondent inferences were stronger for the robot than for the human. In other words, judges of the robot drew a stronger unit relation between the actor and its actions, as evidenced by the larger effect size of popular or unpopular behavior on attributed attitudes for the robot. With unpopular behavior, specifically, judges held the robot more dispositionally culpable than the human. Judges also felt greater confidence in their judgments of the robot’s true attitudes compared to the human.

Research question 2 asked if the nature of the agent’s behavior as popular or unpopular would influence causal attributions and global impressions. The relatively stronger correspondence bias toward robots was driven by the greater dispositional culpability attributed to robots committing unpopular behavior, whether freely chosen or coerced. Participants generally formed more favorable impressions of human versus robot agents and popular behavior versus unpopular behavior. Humans were rated more favorably for popular behavior than for unpopular behavior, regardless of whether they chose or were assigned the behavior.

When forming impressions of robots, there were some differences. For robots committing popular behavior, the same attitudes were attributed to them whether they chose or were assigned. However, judges were more generous in their impressions of the robot when its unpopular behavior was coerced rather than chosen, a tendency not displayed when forming impressions of the human agent. Although judges formed different impressions of the robot that chose to commit popular or unpopular behavior, coerced behavior type had no marked influence on impressions. Paradoxically, people held the robot more dispositionally responsible for its forced unpopular behavior than its chosen unpopular behavior, but were also more generous in their global impressions of the robot when its unpopular behavior was forced. Although judges formed different and valence-congruent impressions of the robots that chose popular or unpopular behavior, the impressions they formed of robots coerced to commit popular or unpopular behavior did not differ.

### 5.2 Implications

First, there were similarities in how participants made causal attributions about robot and human behavior. They made correspondent inferences for both, attributing the cause of behavior to the agent’s disposition even when the agent was coerced to do it. This may support the CASA Paradigm (Reeves and Nass, 1996) by showing similarities in how we treat social robots and people, consistent with prior research drawing parallels in terms of robot mind ascription, intention, goals, and so on. However, the differences between how participants judged humans and robots are perhaps more interesting and important. At the broadest level, these differences in how a classic social psychology finding applied to robots versus humans adds to a small set of studies challenging the notion that people necessarily interpret and react to social robots as they do to other humans. For instance, in an HRI replication of The Milgram Shock Experiment (Bartneck et al., 2005), found that every participant was willing to administer to a robot the highest voltage shock, whereas 60% of participants in the original study refused to use the maximum setting on another human. Furthermore, there are documented differences in the expectations for interaction people hold of social robots versus humans (Spence et al., 2014; Edwards et al., 2016; Edwards et al., 2019) and in their ontological understandings of these agents (Kahn et al., 2011; Edwards, 2018). Results of this experiment are also consistent with the idea that people view robots as unique from humans on dimensions including social presence, agency, free will, status, and capacity for suffering, which may lead them to develop and apply media-centric scripts developed specifically for cognition and behavior toward social robots (Gambino et al., 2020). Although both computer-based technologies and humans may be social actors (CASA), they are not necessarily seen as the same type of social actor.

The question becomes, what is the significance of the specific differences observed in this experiment: 1) that there were stronger dispositional correspondent inferences (stronger actor/agent conflation) for robots than for humans, 2) that people were more certain about a robot’s “true disposition” than a human’s, and 3) that people uncoupled attributed attitudes from global impressions to a greater degree for robots? Satisfying answers will depend upon why people (appeared to) not only commit the fundamental attribution error with robots–which are machines logically understood to operate without interior “dispositions” like personality, attitudes, beliefs, and feeling–but also to commit it to a greater degree and with greater certainty then they did with humans. At first glance, these causal inferences of robot behavior may appear to be a mistake or error akin to the one people make in judging one another.

However, there are three problems with calling the observed results an instance of Fundamental Attribution Error (FAE). The first two arise from cross-application of criticism surrounding human FAE studies using attributed attitude paradigms: 1) the judge never really knows whether the coerced actor actually agrees or disagrees with the direction of their forced action, which means a dispositional attribution is not necessarily incorrect/erroneous and 2) correspondent inferences in which an actor is presumed to possess action-congruent attitudes do not necessarily mean that the central underlying premise of FAE—that people routinely overemphasize dispositional and underemphasize situational causes of behavior—has been supported. These critiques have resulted in a preference for the terms “correspondence bias” or “dispositional correspondent inferences” over FAE when there is no direct test of Situation Theory (S-Theory) awareness and its role in attribution processes (Gawronski, 2004). In the case of robots, there is a third and obvious reason to hesitate to apply the term “error” to a tendency to infer that a robot’s behavior corresponds to its disposition: Logically, it does not seem possible that robots, as programmed machines, hold true dispositions, beliefs, or attitudes that are incongruent with their actions. This is because beliefs and attitudes are widely understood to require inward experiential aspects or subjectivity of thought that does not characterize present robots.

Therefore, viewing the results through the lens of the correspondence bias is more fruitful because it removes both the evaluative aspect of whether people are “right” or “wrong” to conflate a robot agent and its actions and the necessity of linking observed effects to a broad and pervasive underestimation of situational influence, to center only on whether people bend toward disposition-situational convergence. Now the issue remains of how to interpret the relatively stronger and more confident correspondence bias people exhibited toward social robots. As discussed by Gawronski (2004), the correspondence bias may arise from a number of different processes involving how people apply causal theories about the role of situation on behavior (S-Theory). These include 1) lack of S-Theory (when there is no awareness of or there is disagreement with the premise that situational factors constrain behavior), 2) failed application of S-Theory (when there is knowledge of and belief in S-Theory adequacy, but people are unmotivated, lack cognitive capacity, or have inferential goals which result in failure to correct dispositional attribution bias), 3) deliberate neglect of S-Theory (when S-Theory is deemed irrelevant because observed behavior seems highly diagnostic irrespective of situational forces, as in cases of morality and performance ability), and 4) biasing application of S-Theory (when S-Theory is applied in a manner that amplifies rather than attenuates correspondent dispositional inferences) (Gawronski, 2004).

This fourth and final cause of correspondence bias—biasing application of S-Theory—seems especially relevant to understanding why people may make stronger correspondent dispositional inferences for robots than for other humans. The “over-” or biasing application of S-Theory (where “over” implies an attributional effect and not a normative or judgmental inadequacy) may occur in cases in which people understand that behavior is constrained by situational factors, are aware of present situational factors (e.g., whether the behavior was freely chosen or assigned, and the nature of the agent), have the capacity and motivation to apply S-theory, then do so to such a high degree that it appears as if they have totally ignored the causal role of situational factors (Gawronski et al., 2002). For example, people may disambiguate ambiguous human behavior by defining disposition completely in terms of the situation; Ambiguous behavior has been attributed to dispositional anxiety because the situation was perceived as anxiety-inducing (Snyder and Frankel, 1976).

Theoretically, people’s ideas about what robots are, how they work, and how they compare to humans could also lead to a biasing application of S-Theory. To the degree that robots are understood as programmed and controlled by humans, the situation may become salient to the degree it is considered completely determinative of and the same thing as disposition (they are programmed, hence their behavior literally is their personality/attitude/disposition). Ironically, this strong or complete application of S-Theory would appear in the data as heightened dispositional inference because participants would presume alignment between the robot’s behavior and its true attitudes or personality. In reality, this pattern of findings may simply reflect participants’ tendency to conflate an agent whose nature is to lack independent, interior life with its situationally determined actions.

Perhaps most significantly for theorizing HRI, this possible explanation prompts serious consideration of the idea that people may use different causal attribution processes to display a correspondence bias with robots than they do with other humans, even under the same circumstances. Both the stronger and more certain unit relation participants drew between a robot actor and its actions and the looser relationship they displayed between attributed attitudes and general impressions of the robot (i.e., the greater impression-related generosity for robots coerced to do unpopular things compared to humans) compel further investigation into whether unique perceptual patterns and theoretical mechanisms underlie causal inferences of robot behavior. Naturally, people’s causal theories about the role of situation on behavior (S-Theory) may be different for robots and human beings because people perceive them to be ontologically distinct (Kahn et al., 2011; Edwards, 2018).

The FAE, from which correspondence bias research derived, has been called the conceptual bedrock of the field of social psychology, which rests on the assumption that we tend to see others as internally motivated and responsible for their own behavior (Ross, 1977). Drawing a distinction between personality and situation is meaningful when making sense of other humans, and it appears to factor prominently in the dispositional correspondent inferences we tend to make of one another. But with robots, the similar-appearing, but stronger correspondence bias demonstrated by participants could arise from a different psychology altogether, and one more akin to the analytical/logical behaviorism which equates behavioral and mental tendency. Viewed from this lens, much of our descriptive vocabulary for human beings—mind, personality, intention, disposition, attitude, belief—may still be productively transferred to robots, but meant in a different sense [see, e.g., (De Graaf and Malle, 2019)]. Thellman et al. (2017) suggest a similar explanation of their finding that when asked explicitly, people rated goals and dispositions as a more plausible cause of behavior when the actor was human: “This raises the question whether people think of robots as less likely to have dispositions in the human sense, or as having less stable dispositions as humans, or whether people see robot dispositions as less efficacious in causing behavior than human dispositions” (Thellman et al., 2017, p. 11).

Our participants readily attributed to the robot a “true” or “real” attitude and they inferred the nature of that attitude heavily from observed behavior. However, is a robot attitude the same thing as a human attitude (see Nilsson, 2014, on robot “belief”)? Or, is the latter understood to be held (and therefore possibly concealed or subordinated), while the former is purely beheld (manifest, observed, perceived through sight or apprehension), rendering the causal distinction between an agent and its action unhelpful or illogical in the case of robots?

In other words, might people be social psychologists when it comes to other humans and behavioral psychologists when it comes to robots? For commentary on the application of behaviorist principles to robots, see: Sætra (2021); Danaher (2019).

Naturally, working out the fruitfulness of the paths of inquiry suggested above will require asking people what they think about the meaning of attitudes, beliefs, or personality (and situation) in the context of robots, and observing their language and behavior both *in situ* and in experiments designed specifically to test alternative explanations for a correspondence bias (or “agent-action conflation bias”) in HRI and to chart the boundaries of when, where, why, and how it may converge or diverge from human-centric causal inference processes.

In terms of methodological implications for the study of HRI, this research demonstrates the value of including within HRI experiments a human-human condition. Classically, research undertaken within the CASA framework encourages choosing a social science finding (theory and method) that applies to human interaction, replicating the research while substituting a robot/computer for a human actor in the statement of theory and design, providing the robot with human-linked characteristics, and determining whether and to what degree the same social rule still applies (Nass et al., 1994). We argue that including a human-human comparison group offers three advantages to the traditional methodology: 1) it tests again the applicability of the theory to human behavior, which is important given recent replication and reproducibility difficulties in social fields (Maxwell et al., 2015), 2) allows for the identification of both similarities and differences in HHI and HRI (including effect magnitudes) without relying on comparisons between dissimilar datasets and samples, and 3) opens examination of the possibility that even patterns of similarity in HHI and HRI may manifest for a different reason than the mindless application of human social scripts to interactions with robots (Edwards et al., 2019; Gambino et al., 2020; Fortunati and Edwards, 2021). This latter point is especially crucial because the original procedure to conduct CASA research is not sensitive to the potential operation of different theoretical mechanisms responsible for similar observational endpoints. Had we not included a human condition in this experiment, the results would have appeared only to generally mirror a tendency found in human interaction (Forgas, 1998); to suggest people also overemphasize personality at the expense of situational consideration when explaining robot behavior) and left unaddressed questions including “Are we certain the correspondence bias would be replicated with humans today, in this historical and cultural context?” “Are there any differences in how our participants would have evaluated human beings performing the same actions in the same situation?” and “Do any differences, large or small, suggest the possibility that even observed similarities warrant interpretive scrutiny?”.

### 5.3 Future Research

The current study demonstrates that the correspondence bias extends to human-robot interactions. We do not know what factors influence the situational and dispositional attributions people make about robots. Do people over-apply situational theory to robots? In other words, how can bias attenuation occur in an interaction? Identifying future research needs to examine, through experimental design, why exactly people appear to make stronger correspondent inferences for robots than humans and how that will translate to the assignment of credit, blame, moral agency, and moral patiency. Additionally, future research needs to examine what factors may enhance or attenuate correspondent inferences.

People have an anthropocentric bias about conversations in that they expect to speak with a human and not a machine partner. In these studies, people report lower liking for social robots and have greater uncertainty about the interaction (Spence et al., 2014; Edwards et al., 2016; Edwards et al., 2019). Do these findings impact potential attributional errors with social robots? And if so, what can be done to attenuate them? Does the greater uncertainty cause the over-application? Aspects of the robot, including morphology, scripting, interaction modality, and interaction history, should be explored for potential effects on causal attributions of its behavior. Future research needs to explore why people held the robot more dispositionally responsible than the human and why they felt greater confidence in their judgments of the robot’s attitudes than the human actor.

Third, how exactly is responsibility handled differently with robots than humans? Because participants were relatively more kind in their reported impressions of the robot when its bad behavior was coerced (not so for the human agent), we need future research to examine how responsibility for decision-making will occur. Previous research has demonstrated that even when participants are given transparent details about robot behaviors and drives, they thought the robot was thinking more (Wortham et al., 2017). Although it is possible that the meaning of robot “thinking” shifted following explanations of how the robot functioned. We suspect that interpersonal relationship dimensions will come into play. If we have a relationship with a social robot, do we offer more responsibility for decision-making to the robot? We certainly do with people, and it stands to reason that relationships will make a difference in HRI. In the current study, the exposure time was the same for each condition and yet the robot was held more dispositionally responsible. Future research needs to examine if relationship differences can attenuate these differences.

Finally, it is possible that the video stimulus was not as “real-world” as a study with face-to-face embodied presence with the robot. Furthermore, the scenario was hypothetical and pertained to a single, short speech. Potentially, attribution processes play out differently following longer-term, real-world observation of robot behavior, and could differ when evaluating message behavior versus other types. Future research should replicate this study in a live interaction. Being in the room with a social robot might cause a differing correspondence bias than simply watching one on a video. Issues such as social presence (Short et al., 1976) might impact these judgments.

## 6 Conclusion

This study demonstrates that people do exhibit the correspondence bias with social robots. This experiment shows a stronger correspondence bias toward social robots than humans, or the tendency to conflate an agent and its actions into a single categorical unit. Especially in the case of unpopular behavior, judges inferred the robot had more congruent underlying attitudes than the human. The tendency to believe that what people do reflects who they are may be magnified in HRI to the degree that people think what robots do is who they are. People held robots more dispositionally responsible for their unpopular behavior, and people were more confident in their attributions of a robot than human attitudes. Although participants attributed behavior congruent beliefs to robots as they did to other humans, they perhaps did not attribute the possibility of true thoughts incongruent with their actions. As such, we may be social psychologists when interpreting other people and behaviorists when interpreting robots.

## Data Availability

The raw data supporting the conclusion of this article will be made available by the authors, without undue reservation.

## References

[B1] Banks J. Edwards A. P. Westerman D. (2021). The Space between: Nature and Machine Heuristics in Evaluations of Organisms, Cyborgs, and Robots. Cyberpsychology, Behav. Soc. Networking 24, 324–331. 10.1089/cyber.2020.0165 33416419

[B2] Banks J. (2020). Theory of Mind in Social Robots: Replication of Five Established Human Tests. Int. J. Soc. Robotics 12, 403–414. 10.1007/s12369-019-00588-x

[B3] Barrett J. L. (2007). Cognitive Science of Religion: What Is it and Why Is it? Religion Compass 1, 768–786. 10.1111/j.1749-8171.2007.00042.x

[B4] Barrett J. L. (2000). Exploring the Natural Foundations of Religion. Trends Cognitive Sciences 4, 29–34. 10.1016/s1364-6613(99)01419-9 10637620

[B5] Bartneck C. Nomura T. Kanda T. Suzuki T. Kennsuke K. (2005). “A Cross-Cultural Study on Attitudes towards Robots,” in HCI International Conference, Las Vegas, NV, July 2005.

[B6] Berry D. M. Frederickson J. (2015). The Postdigital Constellation. J. Integrated Soc. Sci. 5, 44–57. 10.1057/9781137437204_4

[B7] Branyon J. Pak R. (2015). “Investigating Older Adults' Trust, Causal Attributions, and Perception of Capabilities in Robots as a Function of Robot Appearance, Task, and Reliability,” in Proceedings of the Human Factors and Ergonomics Society Annual Meeting, Los Angeles, CA (Los Angeles, CA): SAGE Publications Sage CA) v.59(1), 1550–1554. 10.1177/1541931215591335

[B8] Ciardo F. Beyer F. De Tommaso D. Wykowska A. (2020). Attribution of Intentional agency towards Robots Reduces One's Own Sense of agency. Cognition 194, 104109. 10.1016/j.cognition.2019.104109 31675616

[B9] Danaher J. (2019). The Philosophical Case for Robot friendship. J. Posthuman Stud. 3, 5–24. 10.5325/jpoststud.3.1.0005

[B10] Daniller A. (2019). Two-thirds of Americans Support Marijuana Legalization. Pew Research Center.

[B11] De Graaf M. M. Malle B. F. (2017). “How People Explain Action (And Autonomous Intelligent Systems Should Too),” in 2017 AAAI Fall Symposium Series, October 2017.

[B12] De Graaf M. M. Malle B. F. (2019). “People’s Explanations of Robot Behavior Subtly Reveal Mental State Inferences,” in 2019 14th ACM/IEEE International Conference on Human-Robot Interaction (HRI) (IEEE), 239–248. 10.1109/hri.2019.8673308

[B13] Edwards A. (2018). “Animals, Humans, and Machines: Interactive Implications of Ontological Classification,” in Human-machine Communication: Rethinking Communication Technology and ourselves (New York, NY: Peter Lang), 29–50.

[B14] Edwards A. Edwards C. Gambino A. (2020). The Social Pragmatics of Communication with Social Robots: Effects of Robot Message Design Logic in a Regulative Context. Int. J. Soc. Robotics 12, 945–957. 10.1007/s12369-019-00538-7

[B15] Edwards A. Edwards C. Westerman D. Spence P. R. (2019). Initial Expectations, Interactions, and beyond with Social Robots. Comput. Hum. Behav. 90, 308–314. 10.1016/j.chb.2018.08.042

[B16] Edwards C. Edwards A. Spence P. R. Westerman D. (2016). Initial Interaction Expectations with Robots: Testing the Human-To-Human Interaction Script. Commun. Stud. 67, 227–238. 10.1080/10510974.2015.1121899

[B17] Forgas J. P. (1998). On Being Happy and Mistaken: Mood Effects on the Fundamental Attribution Error. J. Personal. Soc. Psychol. 75, 318–331. 10.1037/0022-3514.75.2.318 9731311

[B18] Fortunati L. Edwards A. P. (2021). Moving Ahead with Human-Machine Communication. Human-Machine Commun. 2, 1. 10.30658/hmc.2.1

[B19] Gambino A. Fox J. Ratan R. A. (2020). Building a Stronger Casa: Extending the Computers Are Social Actors Paradigm. Human-Machine Commun. 1, 5. 10.30658/hmc.1.5

[B20] Gawronski B. Alshut E. Grafe J. Nespethal J. Ruhmland A. Schulz L. (2002). Prozesse der Urteilsbildung über bekannte und unbekannte Personen. Z. für Sozialpsychologie 33, 25–34. 10.1024//0044-3514.33.1.25

[B21] Gawronski B. (2004). Theory-based Bias Correction in Dispositional Inference: The Fundamental Attribution Error Is Dead, Long Live the Correspondence Bias. Eur. Rev. Soc. Psychol. 15, 183–217. 10.1080/10463280440000026

[B22] Gilbert D. T. Jones E. E. (1986). Perceiver-induced Constraint: Interpretations of Self-Generated Reality. J. Personal. Soc. Psychol. 50, 269–280. 10.1037/0022-3514.50.2.269

[B23] Haring K. S. Watanabe K. Velonaki M. Tossell C. C. Finomore V. (2018). FFAB-the Form Function Attribution Bias in Human-Robot Interaction. IEEE Trans. Cogn. Dev. Syst. 10, 843–851. 10.1109/tcds.2018.2851569

[B24] Hegel F. Muhl C. Wrede B. Hielscher-Fastabend M. Sagerer G. (2009). “Understanding Social Robots,” in 2009 Second International Conferences on Advances in Computer-Human Interactions, Cancun, Mexico, February 2009 (IEEE), 169–174. 10.1109/achi.2009.51

[B25] Heider F. Simmel M. (1944). An Experimental Study of Apparent Behavior. Am. J. Psychol. 57, 243–259. 10.2307/1416950

[B26] Heider F. (1958). The Naive Analysis of Action. New York, NY: Wiley.

[B27] Jones E. E. Harris V. A. (1967). The Attribution of Attitudes. J. Exp. Soc. Psychol. 3, 1–24. 10.1016/0022-1031(67)90034-0

[B28] Jones E. E. (1979). The Rocky Road from Acts to Dispositions. Am. Psychol. 34, 107–117. 10.1037/0003-066x.34.2.107 484919

[B29] Kahn P. H. Reichert A. L. Gary H. E. Kanda T. Ishiguro H. Shen S. (2011). “The New Ontological Category Hypothesis in Human-Robot Interaction,” in 2011 6th ACM/IEEE International Conference on Human-Robot Interaction (HRI), Lausanne, Switzerland, March 2011 (IEEE), 159–160. 10.1145/1957656.1957710

[B30] Langdridge D. Butt T. (2004). The Fundamental Attribution Error: A Phenomenological Critique. Br. J. Soc. Psychol. 43, 357–369. 10.1348/0144666042037962 15479535

[B31] Martini M. C. Gonzalez C. A. Wiese E. (2016). Seeing Minds in Others - Can Agents with Robotic Appearance Have Human-like Preferences? PloS one 11, e0146310. 10.1371/journal.pone.0146310 26745500 PMC4706415

[B32] Maxwell S. E. Lau M. Y. Howard G. S. (2015). Is Psychology Suffering from a Replication Crisis? what Does “Failure to Replicate” Really Mean? Am. Psychol. 70, 487–498. 10.1037/a0039400 26348332

[B33] Nass C. Steuer J. Tauber E. R. (1994). “Computers Are Social Actors,” in Proceedings of the SIGCHI conference on Human factors in computing systems, Boston, Mass, April 1994, 72–78. 10.1145/191666.191703

[B34] Nilsson N. J. (2014). Understanding Beliefs. Cambrige: MIT Press.

[B35] Norenzayan A. Nisbett R. E. (2000). Culture and Causal Cognition. Curr. Dir. Psychol. Sci. 9, 132–135. 10.1111/1467-8721.00077

[B36] Pak R. Crumley-Branyon J. J. de Visser E. J. Rovira E. (2020). Factors that Affect Younger and Older Adults' Causal Attributions of Robot Behaviour. Ergonomics 63, 421–439. 10.1080/00140139.2020.1734242 32096445

[B37] Reeves B. Nass C. (1996). The media Equation: How People Treat Computers, Television, and New media like Real People. Cambridge, UK: Cambridge University Press.

[B38] Riggio H. R. Garcia A. L. (2009). The Power of Situations: Jonestown and the Fundamental Attribution Error. Teach. Psychol. 36, 108–112. 10.1080/00986280902739636

[B39] Robinson J. McArthur L. Z. (1982). Impact of Salient Vocal Qualities on Causal Attribution for a Speaker's Behavior. J. Personal. Soc. Psychol. 43, 236–247. 10.1037/0022-3514.43.2.236

[B40] Ross L. D. Amabile T. M. Steinmetz J. L. (1977). Social Roles, Social Control, and Biases in Social-Perception Processes. J. Personal. Soc. Psychol. 35, 485–494. 10.1037/0022-3514.35.7.485

[B41] Ross L. (1977). The Intuitive Psychologist and His Shortcomings: Distortions in the Attribution Process. Adv. Exp. Soc. Psychol. 10, 173–220. Elsevier. 10.1016/s0065-2601(08)60357-3

[B42] Sætra H. S. (2021). Robotomorphy. AI and Ethics, 1–9. 10.1007/s43681-021-00092-x

[B43] Sciutti A. Bisio A. Nori F. Metta G. Fadiga L. Sandini G. (2013). Robots Can Be Perceived as Goal-Oriented Agents. Is 14, 329–350. 10.1075/is.14.3.02sci

[B44] Short J. Williams E. Christie B. (1976). The Social Psychology of Telecommunications. Toronto; London; New York: Wiley.

[B45] Snyder M. L. Frankel A. (1976). Observer Bias: A Stringent Test of Behavior Engulfing the Field. J. Personal. Soc. Psychol. 34, 857–864. 10.1037/0022-3514.34.5.857

[B46] Spence P. R. Westerman D. Edwards C. Edwards A. (2014). Welcoming Our Robot Overlords: Initial Expectations about Interaction with a Robot. Commun. Res. Rep. 31, 272–280. 10.1080/08824096.2014.924337

[B47] Taylor S. E. Fiske S. T. (1975). Point of View and Perceptions of Causality. J. Personal. Soc. Psychol. 32, 439–445. 10.1037/h0077095

[B48] Thellman S. Silvervarg A. Ziemke T. (2017). Folk-psychological Interpretation of Human vs. Humanoid Robot Behavior: Exploring the Intentional Stance toward Robots. Front. Psychol. 8, 1962. 10.3389/fpsyg.2017.01962 29184519 PMC5694477

[B49] Walster E. (1966). Assignment of Responsibility for an Accident. J. Personal. Soc. Psychol. 3, 73–79. 10.1037/h0022733 5902079

[B50] Wortham R. H. Theodorou A. Bryson J. J. (2017). “Improving Robot Transparency: Real-Time Visualisation of Robot Ai Substantially Improves Understanding in Naive Observers,” in 2017 26th IEEE international symposium on robot and human interactive communication (RO-MAN), Lisbon, Portugal, August 28–September 1, 2017 (IEEE), 1424–1431. 10.1109/roman.2017.8172491

[B51] Ziemke T. Thill S. Vernon D. (2015). “Embodiment Is a Double-Edged Sword in Human-Robot Interaction: Ascribed vs. Intrinsic Intentionality,” in Proceedings of the workshop on cognition: A bridge between robotics and interaction, Portland, 9–10.

